# Examining coordination and equilibrium: an analysis of supply index and spatial evolution characteristics for older adult services in Zhejiang Province

**DOI:** 10.3389/fpubh.2023.1222424

**Published:** 2023-10-05

**Authors:** Hao Ji, Yingying Yu

**Affiliations:** Hangzhou Medical College, Hangzhou, China

**Keywords:** older adult services, service supply, index evaluation system, dynamic evolution, spatial differences

## Abstract

**Objective:**

This study aims to analyze the spatial distribution and dynamic evolution of older adult service supply in Zhejiang Province from 2010 to 2019. Additionally, this research seeks to propose an optimized resource allocation strategy for older adult care services, promoting regional fairness and coordinated development.

**Methods:**

To evaluate the older adult service supply capacity, this research first constructed an evaluation index system based on the Chinese modernization development pattern. Then, an empirical analysis was carried out using a combination of the entropy-TOPSIS method, kernel density estimation, Markov chain analysis, Dagum Gini coefficient, and panel regression model.

**Results:**

The results show an overall upward trend in the supply and service capacity of older adult care in the whole province. However, the spatial distribution of older adult service supply capacity in Zhejiang Province still exhibits a gradient effect, even in the most recent year of 2019. Furthermore, the supply capacity of older adult services shifted to a higher level in the whole province, and regions with high supply capacity had a positive spillover effect on adjacent regions. The overall difference in the older adult service supply capacity of the province showed a decreasing trend. The level of economic development, urbanization rate, transportation capacity, the level of opening up, and the proportion of employees in the tertiary industry had a significant impact on the supply capacity and spatial difference of older adult services.

**Conclusion:**

From the findings, this study puts forth countermeasures and suggestions to optimize the spatial distribution of older adult care services. This includes giving full play to the regional spatial linkage effect, promoting new-type urbanization construction, upgrading the transportation network, and expanding the opening up of the industrial structure. By implementing these measures, a more equitable and coordinated older adult services system can be developed in Zhejiang Province.

## 1. Introduction

Population aging has emerged as an intrinsic national condition and a consequential future trajectory in the social development of China. The seventh National Census reveals that the number of individuals aged 60 years and above in China has surpassed a staggering 260 million, constituting 18.7% of the total population (1). Moreover, a report by the Development Research Center of The State Council highlights the projected decline in the proportion of the working-age population to 64.1 and 56% by 2035 and 2050, respectively, thus engendering a grave imbalance in the age composition of the population. Particularly in the economically prosperous coastal provinces, the older adult cohort demonstrates substantial growth, precipitating a complex and severe predicament pertaining to population aging. Taking Zhejiang Province as a paradigmatic case, the most recent data reveal that the number of individuals aged 60 years and above has reached a considerable 13.29 million, accounting for 24.37% of the entire population by the end of 2022. Consequently, the province has attained a notable degree of moderate aging, emerging as one of the earliest provinces to navigate the intricacies of the aging process. The increasingly stringent contours of population aging will invariably engender a steady expansion of aging-related disabilities, as well as incidences of dementia, within the older adult demographic. The escalating severity of population aging will contribute to a persistent escalation in age-related ailments such as aging-related disabilities and dementia among the older adult population. This predicament not only poses significant challenges to China’s prevailing long-term care and security systems but also exerts a profound impact on the sustainable development of various sectors including economic growth, government finance, regional development, security and stability, and social harmony.

Hence, the establishment of a comprehensive and multi-tiered older adult care service system, along with an enhanced allocation capacity of older adult care service resources, and the assurance of sufficient, equitable, and coordinated supply of older adult care services across regions, not only form the pragmatic foundation for the government’s implementation of a healthy aging strategy but also constitute a vital prerequisite for fostering coordinated regional development and advancing the Chinese-style modernization agenda. As early as 1996, China introduced the “Law on the Protection of the Rights and Interests of the Older Adult,” which called for the enhancement of the social security system for the older adult and the establishment of diverse forms of medical insurance systems to safeguard their fundamental needs. In 2012, the National People’s Congress amended the law, emphasizing that proactively addressing the challenges posed by population aging is a long-term strategic imperative for the nation. In 2020, responding proactively to the escalating phenomenon of population aging, the Fifth Plenary Session of the 19th CPC Central Committee proposed the development of inclusive older adult care services and the construction of an integrated care system that amalgamates medical and health services. In recent years, China has promulgated a series of policies and regulations, including the “Implementation Opinions on Further Expanding the Supply of Older Adult Care Services and Promoting the Consumption of Older Adult Care Services,” the “National Medium and Long-Term Plan for Actively Responding to Population Aging,” and the “14th Five-Year Plan” for the Development of the National Aging Cause and Older Adult Care Service System. These measures have significantly bolstered the framework, scope, and quality of the country’s older adult care service system.

Zhejiang Province, renowned for its pursuit of common prosperity, has implemented various measures to address the challenges posed by population aging. These measures include the introduction of the “Practical Implementation Opinions for the Older Adult” in 2006 by the Zhejiang Provincial Working Committee on Aging, the issuance of the “Opinions on Deepening and Improving the Construction of the Social Older Adult Care Service System” by the Zhejiang Provincial People’s Government in 2011, and the development of the “Vocational Skills Evaluation Measures for Older Adult Care Workers” in 2019. More recently, in 2022, Zhejiang Province has introduced additional directives such as the “Implementation Plan for the Special Action on Older Adult Health Services,” the “Implementation Opinions on Strengthening the Work of Aging in the New Era” aiming to establish a “Zheli Health Care” gold card, and the “Implementation Opinions on Accelerating the Construction of a Basic Old-Age Service System,” accompanied by the “List of Basic Old-Age Services.”

However, within Zhejiang Province, disparities in social and economic development, as well as variations in public expenditure preferences among counties, have led to discernible spatial imbalances in the provision of older adult care services. For example, the economically developed and geographically advantageous “Hangjiahu Plain” area enjoys a larger quantity and higher quality of older adult care resources. In contrast, the “26 counties in the mountainous area of Zhejiang Province,” constituting approximately 24% of the province’s population and characterized by limited economic capacity, face significant challenges in meeting the needs of their aging population. Just as the unreasonable allocation and management of natural resources can lead to environmental, safety, and other sustainability issues (2), the uneven and uncoordinated allocation of older adult care service resources in the planning and arrangement of social resources also gives rise to a series of pressing problems, including conflicts, discontentment, and social instability (3–5). Moreover, it results in accelerated population decline in economically disadvantaged regions with insufficient endowment resources and excessive population concentration in prosperous areas with abundant endowment resources. Consequently, severe regional development imbalances exacerbate disparities in public services such as education, healthcare, and social security, undermining social equity. Ultimately, these factors adversely impact individuals’ quality of life and overall well-being. Furthermore, the strain on resources and environmental pressures in developed regions leads to increased resource consumption and environmental pollution (6), hindering the establishment of common prosperity demonstration zones and impeding the progress of Chinese-style modernization.

This study focuses on addressing the challenge of spatially uneven distribution of older adult care services, with Zhejiang Province as the research subject. It aims to measure the level of older adult care service supply in the province from 2010 to 2019, analyze the spatiotemporal pattern evolution, and identify the key factors and mechanisms influencing the supply of older adult care services. The findings from this study will have significant theoretical and practical implications for Zhejiang Province in developing and enhancing a multi-level older adult care service system, improving the capacity, fairness, and equality of service provision, and establishing a modern model of older adult care services that align with Chinese characteristics.

## 2. Literature review

### 2.1. Theoretical and conceptual research on the supply of older adult care services

The supply of older adult care services encompasses various aspects of the well-being and daily life of the older adult, including physical and mental health, transportation, housing, entertainment, and medical treatment (7). However, the specific content and focus of these services vary across different periods, regions, and individual needs, leading to diverse conceptualizations among scholars. Early research emphasized the family as the primary provider of older adult care services (8), but with the rise of socialized older adult care models, the family-centered approach has evolved. The supply of older adult care services now involves collaboration between non-professional family care and professional care services from institutions and private companies. Scholars influenced by personalized care models propose a demand-oriented framework, guiding care provision based on the specific needs of the older adult (9). Similar concepts, such as “patient/client-centered health care,” are found in other literature (10, 11). Another perspective focuses on the accessibility of older adult care resources, including home community services, institutional care, and administrative support (12). Some scholars define the supply of older adult care services from an industrial chain perspective, arguing that it should meet the growing demand for older adult care. This “medical care and maintenance” supply chain integrates various elements, such as workers, products, facilities, equipment, medical services, information technology, financing, and care system coordination (13). Recent research redefines the supply of older adult care services at the outcome level, emphasizing a service framework that meets the physiological and psychological expectations of the older adult, considering variables such as living environment, location characteristics, service facilities, government investment, safety, and comfort (14). Moreover, recent studies explore the latest trends in the supply of older adult care services, examining the expansion and transformation of content, objectives, modes, quality, and spatial allocation in the new era of the digital economy (15–17).

Previous literature has examined the concept of older adult care service supply from various perspectives, offering valuable theoretical insights for understanding this article’s focus. Building on these studies, this study asserts that the supply of older adult care services encompasses a comprehensive endeavor that integrates diverse resources such as social security, older adult care service, health support, and emotional comfort. It aims to fulfill the survival, health, development, emotional, value, and belonging needs of the older adult within the framework of a “demand-process-goal for older adult care.”

### 2.2. Measurement research on the supply of older adult care services

In addition to theoretical research on the supply of older adult care services, literature also addresses the measurement of such supply. Initially, studies focused on measuring the supply level using simple indicators. For instance, Idvall et al. (18) used “registered nurses to beds” as a key indicator of hospital quality, while Huang et al. (19) assessed the supply level of older adult care services in a specific Singaporean community by considering the number of older adult care workers. However, researchers have questioned the efficacy of single-indicator measurement, leading to the emergence of studies constructing comprehensive evaluation index systems (20). These systems incorporate various indicators such as human resources (e.g., health technicians, medical personnel, and registered nurses), physical resources (e.g., older adult care institutions, day care beds, and older adult assistance service facilities), and financial resources (e.g., healthcare expenditures, pension levels, and older adult subsidies) (21–23).

Beyond measuring the supply level, studies also examine the fairness in the allocation and utilization of older adult care service resources. Scholars such as Culyer and Wagstaff (24) and Sun and Luo (25) highlight equity as a prerequisite for resource allocation measurement and service utilization evaluation. Liu et al. (26) assessed the fairness of China’s health resource allocation between 2009 and 2013, while a Chinese scholar named Ma and Feng (27) proposed measuring the fairness of community home care services based on distribution equity, procedural equity, interpersonal equity, and information equity. Additionally, the research explores the measurement of spatial distribution and accessibility of older adult care service resources. For example, Wan et al. (28) measured the spatial distribution difference of China’s health service resources using geographical big data. Cheng et al. (29) evaluated the spatial accessibility of residential nursing resources for older adult in Beijing based on population and nursing resource data, and Cheng et al. (30) assessed the accessibility level of healthcare services for older adult in Nanjing using GIS data and a tourism survey dataset.

To enhance the effectiveness of measuring the supply of older adult care services, various methods and calculation models have been developed (31–34). Commonly employed techniques include entropy weight, analytic hierarchy process, and data envelopment analysis for measuring service provision level or efficiency. When assessing the fairness of spatial resource allocation, the Gini coefficient method and Theil index method are widely used, often combined with the Lorentz curve to provide a visual depiction of pension resource fairness (35). Methods such as the two-step floating catchment method, shortest path analysis, buffer analysis, and analysis network process are commonly used to measure the spatial accessibility of older adult care service resources (36). In pursuit of more accurate and comprehensive results, researchers have proposed combining multiple analysis methods or dynamic assessment approaches to achieve multidimensional measurement of resource allocation level, spatial fairness, and accessibility (37–39).

### 2.3. Research on the influencing factors of the supply spatial distribution of older adult care services

In the face of the rapidly growing older adult population and limited spatial resources, it is essential to strategically allocate and plan various older adult service resources, such as personnel, facilities, institutions, equipment, and products, to enhance the quality of older adult care services. Recent studies have delved into the factors that influence the equitable distribution of these services. Three key factors have emerged from the literature.

First, the financial autonomy of local governments is crucial. When local governments can independently generate funds without relying on subsidies from higher or parallel-level governments, they can allocate resources more effectively (40, 41). Inadequate financial autonomy may hamper investment in public services, including older adult care, leading to an unjustifiable distribution of services. Second, external environmental factors play a significant role. Regional economic development, urbanization rate, population density, road infrastructure, industrial structure, and transportation systems have been found to exert significant influence on the spatial distribution of public services, including older adult care (41–44). Moreover, the advent of the digital economy era has introduced new dimensions to business innovation, such as those based on artificial intelligence and blockchain technology (45). Third, policy factors are influential. Studies have used mathematical models to analyze the impact of policy tools on the spatial distribution of older adult care services. Findings suggest that effective government policies and their implementation are essential for ensuring an adequate and balanced service supply. A strategic combination of policies can optimize the allocation of resources and improve the overall effectiveness of older adult care services (46).

In recognition of the importance of equitable spatial allocation, the Chinese government has introduced relevant plans and policies. Initiatives such as the “Thirteenth Five-Year Plan for Promoting Equitable Provision of Basic Public Services” (2017) and the “Fourteenth Five-Year Plan” (2021) emphasize the need for equal access to basic public services. Despite these efforts, spatial disparities in older adult care services persist during the current stage of development characterized by “common prosperity.” Therefore, it is crucial to identify and address the factors that contribute to these disparities. By doing so, a high-quality and efficient modern older adult care service system can be established, meeting the growing demand and ensuring the sustainable development of the economy and society.

In conclusion, the research on the supply of older adult care services has made significant progress in the existing literature. Theoretical studies have focused on defining the concept, mode, content, and objectives of older adult care service supply. Empirically, scholars have developed evaluation index systems and measurement methods to assess the supply level, fairness, and efficiency of basic public services, including older adult care services. However, there are limitations in the current research that need to be addressed, especially considering China’s aging population, widening regional development gap, and the “people-oriented” service concept emphasized in Chinese modernization.

First, the existing index system for measuring the supply level of older adult care services needs further improvement and enrichment. Older adult care services encompass a wide range of social security, medical, and emotional support services. However, the current evaluation indicators are limited, leading to potential measurement biases.

Second, a more comprehensive investigation is needed to understand the evolution of spatial distribution patterns of older adult care service supply. The spatial distribution of older adult care services is a complex process that evolves. Most existing studies only provide cross-sectional analyses at specific time points, neglecting temporal changes and trends. This hampers effective policymaking that takes into account dynamic shifts.

Third, a deeper analysis of the factors influencing spatial distribution disparities in the supply of older adult care services is necessary. Current literature predominantly compares absolute spatial differences, providing a surface-level understanding but failing to uncover the underlying causes accurately. To address these shortcomings, this study empirically examines the spatial distribution pattern, differences, and dynamic trends in the supply of older adult care services in the common prosperity demonstration area of Zhejiang Province from 2010 to 2019. Based on the analysis, the author proposes strategies to optimize the supply capacity and resource allocation of older adult care services.

## 3. Study design

### 3.1. Evaluation index system design

In 2022, The State Council released “The 14th Five-Year Plan for the Development of the National Cause for the Aged and the Older Adult Service System” [State Development (2021) No. 35], highlighting the importance of social security, older adult services, and health support. It emphasized the need to enhance the supply of high-level and equitable resources for older adult services to fully meet the diverse and evolving needs of the older adult population (47). Apart from material services, this study recognizes that many older adult individuals experience various psychological and emotional challenges, such as depression, anxiety, and insomnia following the loss of a spouse or limited social engagement (48). To capture the comprehensive notion of “the supply of older adult services” and ensure objectivity, systematization, and operational feasibility, this study constructs an evaluation index system encompassing four dimensions: “social security, older adult care, health support, and emotional comfort” (as presented in Table 1).

**Table 1:** Evaluation index system of older adult care service supply capacity.

Primary indicators	Secondary indicators	Calculation description	Unit	Weight	Directionality	Inspired
1. Social security system	(1) Basic pension standard	Accessing official data from local departments of human resources and finance.	10,000 CNY	0.052	+	Bali (49) and Nyqvist (50)
	(2) Registration rate of basic medical insurance coverage	Accessing official data from the local Health Commission.	%	0.033	+	Tian et al. (51)
	(3) Expenditure on direct medical assistance during the year	Accessing official data from the local Health Insurance Bureau.	10,000 CNY	0.028	+	Trachtenberg and Manns (52)
	(4) Number of legal aid centers per 1,000 older adults people	(Number of legal aid centers for the older adult within the year)/(Number of permanent residents aged 60 and above in the jurisdiction within the year) × 1,000	Each	0.015	+	Amirgaliev and Nurkatova (53)
	(5) Proportion of older adults receiving older adult subsidies	The sum of the number of older adults receiving subsidies for advanced age, nursing, and older adult care services divided by the number of older adults aged 65 and above.	Person	0.038	+	Liu et al. (26)
2. Older adult care service system	(1) Number of nursing institutions per 1,000 older adults	(Number of nursing institutions within the jurisdiction in a year)/(Number of permanent residents aged 60 and above within the jurisdiction) × 1,000.	Each	0.042	+	Zhang et al. (21) and Liu et al. (54)
	(2) Proportion of nursing beds in older adult care institutions	(Number of nursing beds in older adult care institutions in a year)/(Total number of beds in older adult care institutions in a year).	%	0.068	+	Wang et al. (37)
	(3) Number of public welfare older adult care institutions per 1,000 older adults	(Number of non-profit older adult care institutions in the jurisdiction in a year) / (Number of permanent residents aged 60 and above in the jurisdiction in a year) × 1000.	Each	0.049	+	Hjelmar et al. (55) and Morris (56)
	(4) Total value of community older adult care (mutual aid) facilities	Interview government officials to obtain relevant data.	Ten thousand yuan	0.051	+	Liu et al. (54)
	(5) Life expectancy *per capita*	Accessing official data from the local Health Commission.	Year	0.047	+	Boniol et al. (57)
	(6) Average number of employees in older adult care institutions at the end of the year	Accessing official data from local civil affairs departments.	Person	0.073	+	Hjelmar et al. (55) and Morris (56)
3. Health support system	(1) Number of medical beds in health institutions per 1,000 older adults	Accessing official data from the local Health Commission.	Each	0.088	+	Li et al. (38) and Wang et al. (58)
	(2) Number of certified caregivers per 1,000 older adults	(Number of certified caregivers in the year)/(Number of permanent residents aged 60 and above in the jurisdiction in the year) × 1,000	Each	0.092	+	Boniol et al. (57) and Pu (59)
	(3) Proportion of rehabilitation professionals in the community	Interview government officials to obtain relevant data.	%	0.098	+	Li et al. (23)
	(4) Premature mortality rate of major chronic diseases in older adults	Accessing official data from the local Health Commission.	%	0.036	−	Cao et al. (60)
	(5) Health management rate of the older adult population	Accessing official data from the local Health Commission.	%	0.059	+	Chao et al. (61) and Huang et al. (62)
4. Emotional comfort system	(1) Number of senior colleges per 1,000 older adults	(Number of senior colleges in the jurisdiction in the year)/(Number of permanent residents aged 60 and above in the jurisdiction in the year) × 1,000	Each	0.026	+	Erdenee et al. (63)
	(2) Total area of community parks per 1,000 older adults	(Total area of community parks in the jurisdiction in the year)/(Number of permanent residents aged 60 and above in the jurisdiction in the year) × 1,000	Km^2^	0.038	+	Zhang et al. (64)
	(3) Number of older adult associations organizations per 1,000 older adults	(Number of older adult association organizations in the jurisdiction in the year) / (Number of permanent residents aged 60 and above in the jurisdiction in the year) × 1000.	Each	0.035	+	Shao et al. (65)
	(4) Number of older adult activity stations (centers or activity rooms) per 1,000 older adults	[Number of senior activity stations (centers/rooms) in the jurisdiction in the year]/(Number of permanent residents aged 60 and above in the jurisdiction in the year) × 1,000	Each	0.032	+	Boniol et al. (57) and Shao et al. (65)

Measurement indicators were selected and refined based on the literature review. Indicator weights were calculated using the entropy weight method.

(1) A robust social security system is vital for ensuring a sufficient supply of older adult services, encompassing social assistance, social insurance, and social welfare. Social assistance acts as a safety net, providing essential protection to vulnerable individuals in various areas, including medical support, education, and employment. For older adult, medical assistance is particularly crucial. Social insurance serves as the cornerstone of the modern social security system, covering components such as pension insurance, medical insurance, unemployment insurance, and maternity insurance. Regarding older adult care services, the key components are pension insurance and medical insurance. Social welfare, aimed at enhancing citizens’ standard of living, includes both general provisions such as public education and employment assistance and specific support for groups such as older adult, through measures such as old-age subsidies and legal aid. This study employs five indicators, including “Expenditure on direct medical assistance during the year,” to assess the level of old-age service supply within the social security dimension.

(2) Care services encompass various components, including care institutions, care personnel, and care facilities, all aimed at enhancing the quality of life and functional abilities of the older adult. First, older adult care institutions, such as nursing homes, assisted living facilities, and senior apartments, play a crucial role in delivering quality care services. Key elements within these institutions are “nursing beds” and qualified staff, which are essential for providing high-quality care. Additionally, “public welfare older adult care institutions” serve as important platforms for offering care services to all older adult individuals as part of their livelihood support. Second, community care is a significant model for providing older adult care services, with community-based facilities playing a central role in delivering care services. Finally, we included an average life expectancy indicator to assess the effectiveness of care services. To capture the supply level of care services, this study selects six indicators, including the number of nursing institutions per 1,000 older adult people, at the care services level.

(3) Enhancing the supply of older adult care services relies on the development of comprehensive health support services tailored to the aging population. These services encompass a range of crucial elements, including rehabilitation healthcare, general healthcare, disease treatment, and health management. To evaluate and characterize the effectiveness of older adult care services in delivering rehabilitation and healthcare, this research employs two key indicators: the “Number of medical beds in health institutions per 1,000 older adult people” and the “Proportion of rehabilitation professionals in the community.” These indicators provide insights into the availability and accessibility of rehabilitation services within the older adult care framework. Furthermore, to assess the healthcare capacity of older adult care services, the “Number of certified caregivers per 1,000 older adult people” is employed. In addition, the “Premature mortality rate of major chronic diseases in the older adult” and the “Health management rate of the older adult population” are utilized as metrics to gauge the effectiveness of disease treatment and health management within the realm of older adult care service provision. These indicators collectively offer a comprehensive evaluation of the quality and effectiveness of health support services in older adult care.

(4) Enhancing the mental well-being of the older adult represents a pivotal aspect within the realm of older adult care services. Mental well-being pertains to the provision of emotional solace to older individuals, irrespective of the goal of healing, intending to foster a sense of meaning in life. This objective is achieved through a diverse range of approaches, including educational initiatives, active engagement, entertainment, and participation in various activities. These avenues serve to empower older adults, enabling them to transcend feelings of isolation, anger, anxiety, and fear surrounding mortality. To assess the extent of emotional support encompassed in older adult care services, this study utilizes four indicators: “Number of senior colleges per 1,000 older adult people,” “Total area of community parks per 1,000 older adult people,” “Number of senior association organizations per 1,000 older adult people,” and “Number of older adult activity stations (centers or activity rooms) per 1,000 older adult people.” Collectively, these indicators offer valuable insights into the level of emotional support embedded within the supply of older adult care services.

### 3.2. Methods

#### 3.2.1. The measurement method of the supply of older adult services

The evaluation index system for the supply of older adult services was established according to the latest policy regulations and previous research results, as shown in Table 1. The entropy weight method was applied to calculate the weights of each index. The TOPSIS method was used to assess the supply capacity of older adult services in various sampled counties of Zhejiang Province. The specific process was as follows:

First, construct the weighted normalized matrix, denoted as $Z={\left[z_{ij}\right]}_{m\times n}$ ; second, determine the positive and negative ideal solutions $Z^{+}$ and $Z^{-}$ , where $Z^{+}=\left\{\max z_{ij}|i\in \left[1,m\right];j\in J^{+}\right\}$ , $Z^{-}=\left\{\min z_{ij}|i\in \left[1,m\right];j\in J^{-}\right\}$ , and $J^{+}$ and $J^{-}$ are the sets of positive and negative indicators, respectively; again, calculate the positive and negative ideal solution distances $D_{i}^{+}$ and $D_{i}^{-}$ , where $D_{i}^{+}=\sqrt{\omega_{j}\overset{m}{\underset{j=1}{\sum }}{\left(z_{ij}-z_{j}^{+}\right)}^{2}},i\in \left[1,m\right]$ and $D_{i}^{-}=\sqrt{\omega_{j}\overset{m}{\underset{j=1}{\sum }}{\left(z_{ij}-z_{j}^{-}\right)}^{2}},i\in \left[1,m\right]$ ; finally, calculate the relative proximity, denoted as $C_{i}=D_{i}^{-}/\left(D_{i}^{-}+D_{i}^{+}\right)$ , specifically, the closer the value of $C_{i}$ is to 1, the stronger the supply capacity of older adult services, and vice versa.

#### 3.2.2. Analysis of the evolutionary trends in the supply of older adult services

##### 3.2.2.1. Kernel density estimation

The kernel density estimation (KDE) is a non-parametric method for estimating probability density that uses a peak function to represent the density, allowing for the portrayal of the distribution of examined objects’ location, dynamics, extension, and polarization by an uninterrupted time-varying density profile (66). This method has gained popularity for spatial disequilibrium analysis because of its reduced reliance on models, strong robustness, and accurate portrayal of spatial distribution. In this study, we use kernel density distribution curves to analyze the dynamic characteristics of older adult service supply capacity in Zhejiang Province and compare them across different periods. The position of the graph can reflect the high and low supply capacity of older adult care services, while changes in the height and width of the dominant peak can indicate the trend of absolute differences in supply capacity between counties. We can examine the difference between the counties with high supply capacity and those with low supply capacity by looking at the extension of the distribution pattern, and the degree of polarization of supply capacity is indicated by the number of peaks. The calculation method is presented below:


(1) $$\left\{ \begin{array}{c} f\left(x\right)=\frac{1}{Nh}\overset{N}{\underset{i=1}{\sum }}K\left(\frac{X_{i}-x}{h}\right)\\ K\left(x\right)=\frac{1}{\sqrt{2\pi }}\exp\left(-\frac{x^{2}}{2}\right) \end{array}\right.$$

Where $f\left(x\right)$ is the density function of the random variable x , N is the number of observations (the number of counties), $K\left(\Delta \right)$ is the Gaussian kernel function, $X_{i}$ is the independent and identically distributed observations, x is the mean value, and h is the bandwidth of the distribution curve. It is essential to consider that the choice of bandwidth h in kernel density estimation is critical, as extremely large or small values can introduce bias in characterizing the resulting curve. To address this issue, we conducted several iterations to identify the optimal bandwidth h , which we determined to be 0.05. This selection ensures a balanced representation of the density estimation, enhancing the accuracy and reliability of our results.

##### 3.2.2.2. Markov chain and spatial Markov chains

Considering the limitations of the kernel density estimation curve in capturing the relative changes and probabilities associated with the supply capacity of older adult services within a specific region, this research utilizes a traditional Markov chain methodology to investigate the internal dynamic evolution patterns of older adult service supply capacity in different sampled counties of Zhejiang Province. To be more precise, a first-order homogeneous Markov chain model is developed, and the transition probability matrix is estimated using the maximum likelihood estimation technique, which takes into account the current state and transition probabilities. Through this approach, we can depict the evolutionary trends in the supply of older adult services.

Markov chain model exhibits a special form of motion in a stochastic process that takes the form of a finite set of elements, and the elements in the set are the states of the stochastic process, with the salient feature that the conditional distribution of the state $x_{t}$ in period t is related to the state $x_{t-1}$ in the period t-1 . Assuming that the state of $x_{t}=j$ in period t is j , the elements of the set obey the following relations:


(2) $$P\left\{x_{t}=i|x_{t-1}=i,\;x_{t-2}=i_{t-1},\;\cdots ,\;x_{0}=i_{0}\right\}=P\left\{x_{n}=j|x_{n-1}=i\right\}=P_{ij}$$

In this study, $P_{ij}$ is the transition probability of a county's older adult service supply capacity from type i in the year t to type j in the year t+1 , calculated by the maximum likelihood method, $P_{ij}=n_{ij}/n_{i}$ , where $n_{ij}$ is the number of counties in the observation period from type i in the year t-1 to type j in the year t , and $n_{i}$ is the number of counties in the observation period that are of type i . The probability distribution and changes of each state can be measured by dividing the older adult service supply capacity of Zhejiang Province into N states (i.e., constructing N rows and N columns state transfer probability matrix) during the observation period, and then the dynamic evolution pattern, probability, and trend of older adult service supply capacity during the observation period can be examined. On this basis, the transition direction can be analyzed based on the upgrading, invariance, and reduction of the state of the older adult service supply capacity.

However, traditional Markov chains are limited in revealing the influence of spatial factors on the dynamic transfer of older adult service supply capacity. This study introduces spatial Markov chains for further analysis. The spatial Markov chain can evaluate the influence of geographic factors on the probability of transfer of older adult service supply capacity within a region and reveal the intrinsic relationship between the spatiotemporal evolution trend of the examined object and the geographic factors. Its basic idea is as follows: Based on the spatial weight matrix, the Markov chain transition probability matrix N×N is divided into N groups of N×N transition probability matrices. If the spatial lag type of a region in the t period is N , then $P_{mn}\left(N\right)$ represents the spatial transition probability of the region from type m in the t period to type n . In the specific operation, the significance of the impact of geographical factors on the supply capacity of older adult care services in the region should also be tested.

The commonly used test method is the global Moran’s index test. The global Moran’s index assumes homogeneity among spatial units and is used to analyze the spatial correlation pattern of a particular attribute across the entire region. The formula is as follows:


(3) $$I=\frac{\sum_{i=1}^{n}\sum_{j=1}^{n}\omega_{ij}\left(x_{i}-\bar{x}\right)\left(x_{j}-\bar{x}\right)}{s^{2}=\sum_{i=1}^{n}\sum_{j=1}^{n}\omega_{ij}}$$

In formula (3), $s^{2}=\frac{1}{n}\overset{n}{\underset{j=1}{\sum }}\left(x_{i}-\bar{x}\right)$ denotes the variance of synergy, reflecting the degree of spatial dependence among older adult service supplies. $\omega_{ij}$ represents the weight matrix, which captures the spatial relationships between sampled counties. *n* signifies the total number of counties considered in the analysis. The global Moran’s index, ranging from −1 to 1, is employed to measure the spatial patterns of older adult service supply. A positive value indicates the presence of spatial clustering, suggesting localized concentrations of older adult service provision. Conversely, a negative value suggests spatial dispersion, indicating scattered distributions of service provision. A Moran’s index of 0 suggests that spatial factors have no discernible impact on the supply of older adult services, implying a random spatial distribution. This measure provides valuable insights into the spatial characteristics of older adult service supply.

#### 3.2.3. Analysis of spatial differences in the supply of older adult services and the influencing factors

##### 3.2.3.1. Dagum Gini coefficient and its decomposition by subgroups

In this study, we adopt the Gini coefficient proposed by Dagum (67) and its decomposition method by subgroups to empirically study the spatial disparities in the supply capacity of older adult services in Zhejiang Province. The Dagum Gini coefficient is a widely used measure of income inequality that can be adapted to assess the inequality of any distribution, including the spatial distribution of older adult service supply (67). The formula used to calculate the Dagum Gini coefficient is as follows:


(4) $$G=\frac{\sum_{j=1}^{k}\sum_{h=1}^{k}\sum_{i=1}^{n_{j}}\sum_{r=1}^{n_{h}}\left|x_{ji}-x_{\mathrm{hr}}\right|}{2\gamma n^{2}}$$

In formula (4), n is the number of counties, k is the number of blocks (the blocks in this study are Northern Zhejiang region, Southeastern Zhejiang region, and Southwestern Zhejiang region), $n_{j}$ and $n_{h}$ are the number of counties in blocks j and h , respectively, γ is the average of the supply capacity of older adult services in blocks, and $x_{jh}$ and $x_{\mathrm{hr}}$ are the supply capacity of older adult services in each county in blocks j and h , respectively. Based on the above decomposition idea, G is the sum of $G_{nb}$ , $G_{w}$ , and $G_{t}$ . $G_{nb}$ is the difference between the older adult service supply capacity of blocks j and h , i.e., the degree of contribution of the difference between blocks; $G_{w}$ is the difference between the older adult service supply capacity of blocks j or h , i.e., the degree of contribution of the difference within blocks; $G_{t}$ is the residual term of the cross-influence of the older adult service supply capacity between blocks, i.e., the degree of supervariable density contribution.

##### 3.2.3.2. Panel data model

This study presents a panel data model where the supply capacity of older adult services in each county serves as the dependent variable, while the level of economic development, urbanization rate, transportation capacity, the level of opening up, and the proportion of employees in the tertiary industry serve as the independent variables. The aim is to analyze the factors that influence the spatial differences in the supply capacity of older adult services. The model can be expressed as follows:


(5) $$Y_{\mathrm{it}}=\alpha_{i}+\gamma_{n}\overset{n}{\underset{n-1}{\sum }}X_{itn}+\varepsilon_{\mathrm{it}}$$

In formula (5), i is the number of counties, t is the year distribution, $\alpha_{i}$ is the cross-sectional term of the model, and $\varepsilon_{\mathrm{it}}$ is the random disturbance term of the model; $Y_{\mathrm{it}}$ is the dependent variable, which represents the supply capacity of older adult services in each county; $X_{\mathrm{it}}$ is the independent variable (explanatory variable), and n is the number of independent variables. The variables in the regression model are explained as follows:

###### 3.2.3.2.1. The level of economic development (econ)

Previous studies have consistently demonstrated a positive association between the level of local economic development and the capacity of local governments to deliver public services, particularly in the domain of older adult care (68). Consequently, regions characterized by higher gross domestic product (GDP) are typically endowed with greater governmental resources to support and provide comprehensive older adult services, whereas regions with lower GDP exhibit relatively constrained capacities to meet such service demands. Accordingly, this research adopts GDP as an indicator proxying the level of economic development.

###### 3.2.3.2.2. Urbanization rate (urba)

Previous research has shown that urbanization plays a vital role in overcoming the challenges posed by the urban–rural divide, facilitating economic development, and enhancing coordinated regional growth. It also contributes significantly to the enhancement of the supply capacity of older adult services (69). In this study, the urbanization rate of each county is determined by considering the proportion of the year-end urban population concerning the year-end resident population.

###### 3.2.3.2.3. Transportation capacity (tran)

Based on the principles of new economic geography, efficient transportation networks foster regional connectivity, facilitate the spillover of economic activities across city boundaries and clusters, and give rise to scale effects and scope economies (70). These dynamics provide local governments with opportunities to lower public service costs and improve service provision. In this study, transportation capacity is quantified by calculating the weighted sum of passenger and freight traffic volume, serving as a measure of the region’s transportation infrastructure and its ability to support service delivery.

###### 3.2.3.2.4. The level of opening up (open)

Previous research has established that the extent of economic openness serves as a valuable measure of the local economy’s development status, dynamism, and potential, and has a discernible influence on the capacity to provide older adult services to some extent (71). To capture this aspect, this study adopts the ratio of actual foreign capital utilization to local GDP as an indicator to assess the level of economic openness.

###### 3.2.3.2.5. The proportion of employees in the tertiary industry (empl)

Prior research indicates that the industry is the fundamental unit of regional development, and the size and quality of the tertiary industry are crucial for achieving high-quality economic and social development (72). Improving the concentration level and investment intensity of the tertiary industry in different cities (clusters) is essential for enhancing the supply of public goods and promoting the capacity of older adult care services. The calculation method is the proportion of people engaged in the tertiary industry to the total employed population.

### 3.3. Data source and research description

To investigate the spatial disparities in the supply capacity of older adult services in Zhejiang Province, this study adopts a regional approach, dividing the area into three distinct regions: Northern, Southeastern, and Southwestern Zhejiang. The entropy-TOPSIS method is employed to standardize the data about the older adult service supply capacity in each sampled county of Zhejiang Province. Additionally, panel regression analysis is conducted, utilizing the natural logarithm of relevant variables to address potential issues of non-stationarity and non-linearity. The primary data sources encompass various official statistical publications, including the China Civil Affairs Statistical Yearbook, China County Statistical Yearbook, China Health Statistical Yearbook, China Social Statistical Yearbook, China Transportation Statistical Yearbook, Zhejiang Civil Affairs Development Statistical Bulletin, Zhejiang Statistical Yearbook, and Zhejiang Health Yearbook, alongside statistical yearbooks from 11 prefecture-level cities in Zhejiang Province and publicly accessible statistical bulletins released by the respective prefecture-level city statistical bureaus’ websites. Furthermore, to supplement publicly available data, the research team conducted targeted surveys to collect additional information from relevant government officials, ensuring a comprehensive dataset for analysis.

## 4. Empirical analysis

### 4.1. Dynamic evolution of older adult service supply in Zhejiang Province

#### 4.1.1. Dynamic characterization of kernel density estimation

To capture the dynamic evolution of older adult service supply in different regions of Zhejiang Province, this study utilizes the Gaussian kernel function to estimate the kernel density. This approach enables us to examine the distribution position, situation, extensibility, and polarization trend of the older adult service supply capacity in different periods, allowing for a comprehensive exploration of its dynamic characteristics.

**Figure 1:**
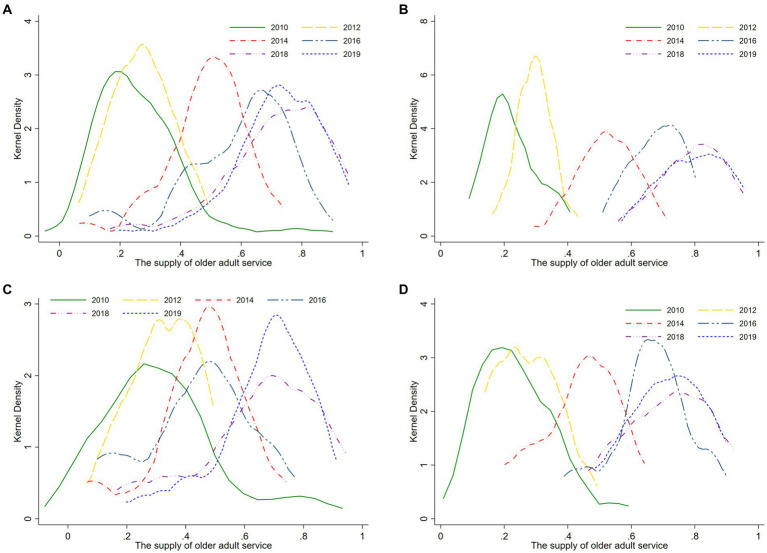
Distribution dynamics of the supply capacity of older adult services in Zhejiang Province from 2010 to 2019. **(A)** Provincial level. **(B)** Northern Zhejiang region. **(C)** Southeastern Zhejiang region. **(D)** Southwestern Zhejiang region.

To reflect the dynamic trend of the older adult service supply index and enhance comparability among different observation datasets, we established 2010 as the baseline and selected observation datasets at a biennial interval. This systematic approach enabled us to generate kernel density estimation curves for older adult service supply in the years 2010, 2012, 2014, 2016, and 2018. Additionally, to capture the latest features of older adult service supply, we incorporated data from 2019 and plotted the corresponding kernel density estimation curve. The specific estimation results are presented in Figure 1.

##### 4.1.1.1. Distribution dynamics at the province-wide level

Figure 1A illustrates the dynamic evolution trend of the supply capacity of older adult services in the province. During the observation period: ① In terms of position, the center axis of the kernel density distribution curve gradually shifts to the right over the years, indicating a steady improvement in the province’s supply capacity of older adult services. ② Regarding the situation, the connecting line of the main peak points of the kernel density distribution curves exhibits a fluctuation pattern resembling the shape of the letter “N.” Furthermore, the width of the curves undergoes a slight narrowing, followed by expansion, and then a subsequent slight narrowing. This implies a reduction in the overall dispersion of the supply capacity of older adult services in the province, with a narrowing trend in the absolute differences between regions. ③ In terms of extensibility, the kernel density distribution curve demonstrates a significant right-tail phenomenon in 2010, gradually transitioning into a “half left distribution” pattern in 2018 and 2019. This indicates an overall upgrade in the supply capacity of older adult services in recent years, accompanied by a moderation of the gap between regions categorized as “high level” and “low level” in terms of supply capacity. ④ With regard to polarization trends, since 2014, the kernel density distribution curve of supply capacity exhibits less pronounced “multi-peak” patterns, with 2016 being the most distinct. Notably, the “dominant peak” corresponding to areas with high supply capacity surpasses the “secondary peak” corresponding to areas with low supply capacity. This suggests a gradient effect in the supply capacity of older adult services across all regions of the province, characterized by bipolar or multipolar differentiation. However, the differentiation trend is not significant, and recent years have shown signs of gradual alleviation.

##### 4.1.1.2. Distribution dynamics at each region level

Figures 1B–D present the dynamic evolution trends of the supply capacity of older adult services in the Northern Zhejiang region, Southeastern Zhejiang region, and Southwestern Zhejiang region, respectively. During the observation period: ① In terms of location, the center positions of the kernel density distribution curves for the supply capacity of older adult services in these three regions shifted to the right as the years increased, indicating a continuous improvement in the level of older adult service supply in the aforementioned areas throughout the observation period. ② Regarding trends, the main peak height of the kernel density curve for older adult service supply in the Northern Zhejiang region showed a fluctuating downward trend, and at the same time, the curve width slightly increased over the years, indicating an expansion of the absolute differences in its older adult service supply capacity. The main peak height of the kernel density curve for older adult service supply in the Southeastern Zhejiang region exhibits an overall fluctuating “N-shaped” trend, and the curve width shows a certain degree of narrowing over time, indicating a gradual reduction in the absolute differences in the older adult service supply capacity in this region. The main peak height of the kernel density curve for older adult service supply in the Southwestern Zhejiang region showed less noticeable changes, and the curve width displayed a slight decrease followed by a slight expansion trend, indicating the process of decreasing and then enlarging absolute differences in its older adult service supply capacity. ③ In terms of extendibility, the kernel density distribution curve in the Southeastern Zhejiang region displayed a significant right-tail phenomenon in the initial years, but as time passed, the tailing phenomenon reduced, indicating a significant improvement in the spatial differences in older adult service supply level during the observation period. In comparison to the Southeastern Zhejiang region, the kernel density distribution curves in the Northern Zhejiang region and Southwestern Zhejiang region did not exhibit significant right-tail phenomena. ④ In terms of polarization trends, the kernel density distribution curves in the Northern Zhejiang region, Southeastern Zhejiang region, and Southwestern Zhejiang region displayed multi-peak or bimodal trends in one or more years, indicating a certain degree of gradient effect in the overall levels of older adult service supply in these regions. However, compared to the Southeastern and Southwestern Zhejiang regions, the gradient effect in the Northern Zhejiang region was the least significant. Finally, the kernel density distribution curves in the Northern Zhejiang region, Southeastern Zhejiang region, and Southwestern Zhejiang region evolved from a “right-skewed distribution” in 2010 to a “left-skewed distribution” in 2018 and 2019, indicating a significant improvement in the supply capacity of older adult services in these regions during the observation period. It also implies a narrowing gap between regions with “high-level” and “low-level” supply capacity, indicating an enhancement in the regional synergy of older adult service supply in Zhejiang Province throughout the entire observation period.

#### 4.1.2. Dynamic characterization of Markov chain

##### 4.1.2.1. Traditional Markov chain

The kernel density distribution curve provides a visual representation of how the supply capacity of older adult services in Zhejiang Province changes over time during the observation period. However, it has limitations in capturing the underlying dynamics of this development comprehensively. To address this issue, the present study employs the Markov chain analysis method, which enables a more in-depth investigation of the transition probabilities and trends in the supply capacity of older adult services across different regions of Zhejiang Province from 2010 to 2019.

To begin with, the quartile method is utilized to classify the measurement results of older adult services supply capacity in each sampled county into four distinct levels. Level I corresponds to a low level (<25%), Level II corresponds to a medium-low level (25 ~ 50%), Level III corresponds to a medium-high level (50 ~ 75%), and Level IV corresponds to a high level (>75%) (for more detailed information, please refer to Table 2).

**Table 2:** Quartile division of measured results for older adult services supply capacity in Zhejiang Province from 2010 to 2019.

Quartile ranges	Obs	Mean	SD	Min	Max
Level I ≤ Q1	153	0.148	0.076	0.001	0.278
Q1 < Level II ≤Q2	152	0.381	0.056	0.281	0.476
Q2< Level III ≤Q3	152	0.573	0.055	0.483	0.663
Q3 < Level IV	153	0.781	0.080	0.665	0.956
Total	610	0.471	0.244	0.001	0.956

The measurement results represent the values of the older adult services supply index for 61 sample counties in Zhejiang Province from 2010 to 2019, ranging between 0 and 1. The first quartile Q1 = 0.2789, the second quartile Q2 = 0.4794, and the third quartile Q3 = 0.6646.

Subsequently, the Markov chain analysis method is applied to calculate the state transition probability matrix of the older adult services supply capacity in Zhejiang Province throughout the observation period, based on the aforementioned classification. The obtained results are presented in Table 3, revealing the probabilities of transitioning between different supply capacity levels over time.

**Table 3:** Markov chain transition probability matrix for older adult services supply capacity in Zhejiang Province from 2010 to 2019.

Type	*n*	I	II	III	IV
I	152	60.53%	32.89%	3.95%	2.63%
II	150	12.67%	46.00%	35.33%	6.00%
III	137	2.19%	5.11%	59.12%	33.58%
IV	110	2.73%	4.55%	9.09%	83.64%

“n” represents the number of counties included in regions of different levels during the observation period. This table includes the same information.

In Table 3, the values on the diagonal line represent the probability of the regional older adult service supply capacity maintaining its state type, indicating the stability of the evolution of the supply capacity. The values on the non-diagonal line represent the probability of the regional older adult services supply capacity transitioning between different state types, providing an intuitive understanding of the evolutionary trend and characteristics of the supply capacity in Zhejiang Province, without considering spatial factors.

First, the diagonal line consistently exhibits higher values than the off-diagonal line, revealing a pattern of higher stability in maintaining the original level of older adult service supply capacity. After 1 year, the probabilities of sample counties in Level I, Level II, Level III, and Level IV maintaining their respective levels are 57.35, 42.65, 50.00, and 81.52%, respectively. This phenomenon suggests a notable “club convergence” effect among different levels.

Second, the probabilities at the ends of the diagonal line are higher than those in the middle, indicating a relatively greater likelihood for sample counties with low-level and high-level older adult service supply capacity to remain at their original levels. This finding highlights a more pronounced club convergence phenomenon for both low-level and high-level supply capacities.

Third, there is an overall significant trend of older adult service supply capacity progressing from low level to high level. The probabilities of transitioning one level up after 1 year for Level I (low level), Level II (medium-low level), and Level III (medium-high level) are 32.89, 35.33, and 33.58%, respectively. Moreover, the results suggest the possibility of leapfrogging the older adult service supply capacity in Zhejiang Province. Specifically, the probabilities of Level I (low level) leaping to Level III (medium-high level) and Level IV (high level) are 3.95 and 2.63%, respectively. The probability of Level II (medium-low level) leaping to Level IV (high level) is 6.00%. However, it is important to note that the probabilities of leapfrogging between levels are lower compared to the probabilities of transitioning between adjacent types.

Additionally, it is worth emphasizing that there is a probability of downward transitions in older adult service supply capacity after 1 year. The probabilities of Level II (medium-low level), Level III (medium-high level), and Level IV (high level) transitioning one level down are 12.67, 5.11, and 9.09%, respectively. This indicates the presence of a certain risk of downward mobility in the older adult service supply capacity in Zhejiang Province, requiring attention and vigilance from relevant governmental and management departments.

##### 4.1.2.2. Spatial Markov chains

Traditional Markov chain analysis, when reflecting the transition characteristics of older adult services supply capacity in different regions of Zhejiang Province, treats each region as an independent unit and does not consider the influence of surrounding neighboring types on its evolution. However, the dynamic progression of older adult services supply capacity, whether upward or downward, based on location factors, is not isolated. Existing research has shown a significant spatial correlation in older adult services supply capacity (73). Therefore, this study incorporates a geographic spatial weight matrix and further verifies the global Moran’s Index of older adult services supply capacity in Zhejiang Province using Formula (3). The results are presented in Table 4. It can be observed that during the observation period, the global Moran’s Index of older adult services supply capacity in Zhejiang Province is consistently positive and significant, indicating a significant positive spatial correlation among the sampled counties in Zhejiang Province in terms of older adult services supply capacity.

**Table 4:** Global Moran’s index of older adult services supply capacity in sampled counties of Zhejiang Province from 2010 to 2019.

Variables	*I*-value	E(I)	SD(I)	*Z-*value	p*
2010 year	0.324***	−0.017	0.096	3.530	0.000
2011 year	0.213**	−0.017	0.099	2.315	0.010
2012 year	0.116*	−0.017	0.100	1.319	0.094
2013 year	0.124*	−0.017	0.100	1.405	0.080
2014 year	0.147***	−0.017	0.099	1.655	0.049
2015 year	0.223***	−0.017	0.100	2.411	0.008
2016 year	0.358***	−0.017	0.099	3.771	0.000
2017 year	0.290***	−0.017	0.099	3.092	0.001
2018 year	0.252***	−0.017	0.099	2.715	0.003
2019 year	0.394***	−0.017	0.098	4.175	0.000

***, **, and * represent significance at the 1, 5, and 10% levels, respectively.

The examination results of the global Moran’s I index indicate that spatial factors have a significant impact on the older adult service supply capacity in sample counties of Zhejiang Province. Therefore, it is necessary to incorporate the “spatial” variable into the research scope and construct a spatial Markov chain probability transition model. The classification criteria for older adult service supply capacity remain consistent with Table 2 mentioned earlier, and the calculation results are presented in Table 5.

**Table 5:** Spatial Markov chain transfer probability matrix of older adult service supply in Zhejiang Province from 2010 to 2019.

Adjacent type	*t*/*t*+1	*n*	I	II	III	IV
I	I	107	67.29%	28.97%	2.80%	0.93%
	II	43	27.91%	53.49%	18.60%	0.00%
	III	5	0.00%	20.00%	60.00%	20.00%
	IV	1	0.00%	0.00%	0.00%	100.00%
II	I	37	45.95%	43.24%	8.11%	2.70%
	II	66	9.09%	45.45%	42.42%	3.03%
	III	42	7.14%	4.76%	66.67%	21.43%
	IV	10	10.00%	10.00%	20.00%	60.00%
III	I	6	50.00%	33.33%	0.00%	16.67%
	II	28	0.00%	42.86%	50.00%	7.14%
	III	68	0.00%	5.88%	52.94%	41.18%
	IV	42	4.76%	9.52%	11.90%	73.81%
IV	I	2	0.00%	50.00%	0.00%	50.00%
	II	13	7.69%	30.77%	23.08%	38.46%
	III	22	0.00%	0.00%	63.64%	36.36%
	IV	57	0.00%	0.00%	5.26%	94.74%

“Adjacent” refers to counties that share a common border.

As shown in Table 5, the influence of spatial effects on the supply capacity of older adult services in Zhejiang Province is mainly manifested as follows:

First, the transfer probability matrices differ under different spatial lag types, indicating that the probability of older adult service supply capacity transfer in this county, influenced by the variations in neighboring counties’ capacities, varies among different spatial lag types.

Second, the diagonal elements of the transfer probability matrices are not always greater than the off-diagonal elements under different spatial lag types. This result suggests that, under spatial spillover effects, the probability of “level lock-in” in older adult service supply capacity is reduced, particularly evident under spatial lag types III and IV.

Furthermore, there are non-zero elements on both sides of the diagonal. This indicates the presence of instability in the older adult service supply among counties in Zhejiang Province. Although upward transitions to an ideal state are possible, there is also a certain risk of downward transitions. Additionally, non-adjacent level transitions are observed.

Moreover, different spatial lag types have varying impacts on the same level. Under spatial lag type III, the probability of transitioning from Level III (medium-high level) to Level IV (high level) is 41.18%, significantly higher than the probability of the same level transition under spatial lag type II, which is 21.43%. This result suggests that regions with higher older adult service supply capacity exhibit positive spillover effects on neighboring areas, demonstrating an overall phenomenon of club convergence.

Finally, the impacts of the same spatial lag type on different levels also differ. For example, under spatial lag type II, the probabilities of upward transitions by one level for Level I (low level), Level II (medium-low level), and Level III (medium-high level) are 43.24, 42.42, and 21.43%, respectively, showing a decreasing trend. This indicates that the transition probabilities are influenced not only by spatial lag types but also by the initial level of older adult service supply capacity.

### 4.2. Analysis of spatial differences in older adult service supply in Zhejiang Province and its underlying causes

#### 4.2.1. Spatial differences in the supply of older adult services

**Figure 2:**
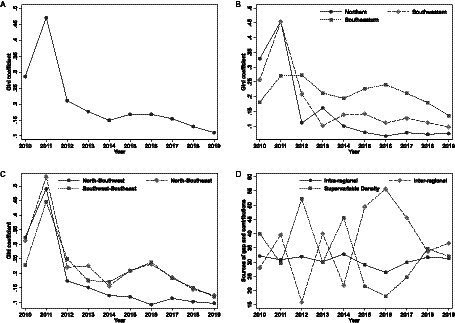
Trend in regional Gini coefficient of older adult services supply capacity in Zhejiang Province. **(A)** Overall Gini coefficient. **(B)** Intra-regional Gini coefficient. **(C)** Inter-regional Gini coefficient. **(D)** Contribution rate.

This study measures and decomposes the regional differences in the supply capacity of older adult services in Zhejiang Province from 2010 to 2019 using the Dagum Gini coefficient, with results presented in Table 6 and Figure 2, to examine the magnitude and sources of the relative differences in the supply capacity of older adult services among regions.

**Table 6:** Regional Gini coefficient and decomposition results of older adult services supply capacity in Zhejiang Province from 2010 to 2019.

Year	Overall	Inter-regional			Intra-regional			Contribution rate(%)		
		North-Southwest	North-Southeast	Southwest-Southeast	Northern	Southwestern	Southeastern	Intra-regional	Inter-regional	Supervariable
2010	0.2859	0.3231	0.3118	0.2273	0.3287	0.2571	0.1814	32.11	27.96	39.94
2011	0.4698	0.4901	0.5317	0.4451	0.4547	0.4534	0.2711	30.83	39.59	29.59
2012	0.2108	0.1735	0.2206	0.2491	0.1116	0.2093	0.2733	31.91	15.89	52.21
2013	0.1765	0.1510	0.2251	0.1751	0.1617	0.1015	0.2120	30.15	39.96	29.89
2014	0.1493	0.1234	0.1564	0.1701	0.1004	0.1384	0.194	32.76	21.71	45.53
2015	0.1677	0.119	0.2088	0.2079	0.0788	0.1416	0.2266	29.03	49.43	21.54
2016	0.1681	0.0921	0.2318	0.2373	0.0667	0.1105	0.2407	26.37	55.66	17.97
2017	0.1537	0.1138	0.1866	0.1836	0.078	0.1268	0.2116	29.87	45.55	24.58
2018	0.1297	0.1021	0.145	0.1499	0.0727	0.1119	0.1795	31.66	33.72	34.61
2019	0.1107	0.0966	0.1244	0.1199	0.0751	0.0975	0.1358	31.32	36.65	32.03
Mean	0.2022	0.1785	0.2342	0.2165	0.1528	0.1748	0.2126	30.60	36.61	32.79

Figure 2A depicts the overall trend of the Gini coefficient for older adult service supply capacity in Zhejiang Province. The coefficient shows a fluctuating downward trajectory throughout the observation period. Notably, it declines from 0.2859 in 2010 to 0.1107 in 2019, marking a substantial reduction of 61.29%. Of particular significance is the average Gini coefficient for older adult service supply capacity, which has decreased since 2015 and reached 0.1460. This decline indicates a consistent narrowing of regional disparities in older adult service supply, contributing to enhanced fairness and balance in service delivery. These positive outcomes can be attributed to the comprehensive implementation of strategic initiatives such as “Healthy Zhejiang,” “Zhejiang’s Health and Wellness in Every Household,” “Urban–Rural Integration Outline,” and “Demonstration Zone for Common Prosperity” since the 18th National Congress. These initiatives have fostered coordinated urban–rural development, efficient allocation of older adult resources, and significant improvements in the accessibility and equity of older adult service supply in Zhejiang Province.

Figure 2B illustrates the intra-regional trends of Gini coefficients for older adult service supply capacity in Zhejiang Province. Throughout the observation period, the Northern, Southwestern, and Southeastern regions experienced a general fluctuating decline in Gini coefficients, reflecting the disparities in older adult service provision. Specifically, the Gini coefficients decreased from 0.3287, 0.2571, and 0.1814 in 2010 to 0.0751, 0.0975, and 0.1358 in 2019 for the Northern, Southwestern, and Southeastern regions, respectively. These reductions amounted to substantial decreases of 77.15, 62.10, and 25.12%. Notably, the Northern region demonstrated the most significant decline, while the Southeastern region exhibited a comparatively smaller decrease. The average Gini coefficients for the Northern, Southwestern, and Southeastern regions were 0.1528, 0.1748, and 0.2126, respectively. These findings further support the patterns revealed by the earlier discussed kernel density estimation curves. In particular, in comparison to the other two regions, the Southeastern region of Zhejiang Province displayed lower fairness in older adult service supply, potentially influenced by the inclusion of underdeveloped areas such as Taishun County, Wencheng County, and remote island mountainous regions.

Figure 2C illustrates the dynamics of inter-regional Gini coefficients for the supply capacity of older adult services in Zhejiang Province. During the observation period, the Gini coefficient trends for older adult service supply among the Northern, Southwestern, and Southeastern regions of Zhejiang Province exhibited a fluctuating downward pattern. After 2015, the Gini coefficient trends for older adult service supply between the Northern and Southeastern regions, as well as between the Southwestern and Southeastern regions, showed a similar pattern. The Gini coefficient levels for older adult service supply between the Northern and Southwestern regions, Northern and Southeastern regions, and Southwestern and Southeastern regions decreased from 0.3231, 0.3118, and 0.2273 in 2010 to 0.0966, 0.1244, and 0.1199 in 2019, respectively, representing reductions of 70.10, 60.10, and 47.27%. These findings indicate a significant improvement in the uneven distribution of older adult service supply among the three major regions of Zhejiang Province during the observation period. However, in terms of mean values, there still exists a significant disparity in older adult service supply capacity between the Northern and Southeastern regions, with a Gini coefficient value of 0.2052, which is noticeably higher than the provincial average. Therefore, it is necessary to accelerate the equalization process of “Zhejiang Healthcare” to meet the older adult care needs of the aging population across the entire province.

Figure 2D presents the evolutionary trajectory of contribution rates to the disparity in older adult service supply capacity in Zhejiang Province. Throughout the observation period, the contribution rates to intra-regional disparity remained relatively steady, fluctuating between 25 and 30%. In contrast, the contribution rates to inter-regional disparity displayed an “M” pattern before 2014, transitioning to a “reverse U” pattern thereafter. They peaked in 2015, 2016, and 2017, playing a significant role in driving the overall regional disparity during that period. Subsequently, these contribution rates rapidly declined, stabilizing at approximately 30% in 2018 and 2019. Conversely, the contribution rates for supervariable density disparity exhibited an inverse relationship with the inter-regional disparity rates. They followed a “W + U” trajectory with 2014 as the turning point. While remaining high in 2012 and 2014, they experienced diminished fluctuations at a lower level from 2015 to 2017. Similarly, they reached a stable state at around 30% in 2018 and 2019. Notably, in the most recent years of 2018 and 2019, intra-regional disparity, inter-regional disparity, and the mutual overlap between regions emerged as equally significant factors contributing to the disparity in older adult service supply.

#### 4.2.2. Causes of spatial differences in the supply of older adult services

This study employs panel data regression models, including the mixed least squares regression model, the random effects model, and the fixed effects model, to conduct regression analysis and robustness tests on the factors influencing the spatiotemporal variations in older adult service supply. The results are presented in Table 7. The B-P LM test indicates that the random effects model outperforms the mixed least squares regression model. The Hausman test demonstrates that the fixed effects model outperforms the random effects model. To mitigate the influence of individual and time-specific factors, this study utilizes a two-way fixed effects model for analysis.

**Table 7:** Regression analysis of factors influencing the supply capacity of older adult services in Zhejiang Province.

Test method	Mixed least-squares model	Random effects model	Fixed effect model
Wald chi^2^(5)	/	482.88***	/
		Prob > chi^2^ = 0.0000	
B-P LM test (chi^2^)	chibar^2^(01) = 114.75***	/	/
	Prob > chibar^2^ = 0.0000		
Hausman test	/	/	chi^2^(5) = 220.67***
			Prob>chi^2^ = 0.0000

Explanatory variable	Coef.	St.Err.	*t*-value	*p*	[95%Conf	Interval]	Sig
Ln(econ)	0.165	0.030	5.50	0.000	0.106	0.223	***
Ln(urba)	0.192	0.044	4.37	0.000	0.106	0.278	***
Ln(tran)	0.295	0.071	4.16	0.000	0.156	0.435	***
Ln(open)	0.094	0.036	2.60	0.001	0.023	0.165	***
Ln(empl)	0.077	0.037	2.10	0.037	0.005	0.149	**
Constant	−8.808	0.523	−16.84	0.000	−9.835	−7.78	***
Mean dependent var	0.470		SD dependent var		0.244		
R-squared	0.667		Number of obs		610		
*F*-test	218.408		Prob > F		0.000		
Akaike crit. (AIC)	−718.449		Bayesian crit. (BIC)		−691.968		

***p < 0.01, **p < 0.05, *p < 0.1.

The analysis presented in the table above reveals the diverse impacts of heterogeneity factors on the supply of older adult services in Zhejiang Province. The key findings are as follows:

(1) Economic development demonstrates a significant positive influence on the supply capacity of older adult care services. A 1% increase in economic development leads to a corresponding increase of 0.165% in service capacity. These results underscore the pivotal role of local economic development in driving the supply of older adult care services.

(2) There is a significant positive correlation between the urbanization rate and the supply of older adult services, with a significance level of 0.01. A 1% increase in the urbanization rate results in a 0.192% increase in service capacity, highlighting the potential of reducing the urban–rural dichotomy and enhancing urbanization to improve the older adult service supply.

(3) Transportation capacity exhibits a significant positive correlation with the supply of older adult services at a significance level of 0.01. Each 1% increase in transportation capacity corresponds to a 0.295% increase in service supply, indicating that a well-developed transportation infrastructure and support system enhance the capacity to provide older adult services.

(4) The level of opening up shows a significant positive correlation with the supply of older adult services at a significance level of 0.01. A 1% increase in the level of opening up leads to a 0.094% increase in service capacity. These findings highlight the role of regional openness in enhancing the supply capacity of older adult services. Therefore, efforts to promote a higher-level open economy system, optimize the regional opening pattern, and enhance the quality of openness can contribute to high-quality economic development and strengthen the government’s capacity to provide public services.

(5) The proportion of employees in the tertiary industry has a significant positive impact on the supply of older adult care services. A 1% increase in this proportion results in a 0.077% increase in service capacity, indicating that policies aimed at optimizing the industrial structure and cultivating talents can effectively enhance the supply capacity of older adult services.

Overall, these findings provide valuable insights for policy formulation by the government, highlighting the importance of economic development, urbanization, transportation capacity, openness, and industrial structure optimization in promoting the capacity and quality of the older adult service supply.

## 5. Conclusion and suggestions

### 5.1. Conclusion

This study defines the connotation of older adult care service supply and subsequently constructs a measurement index system for older adult care service supply based on four dimensions: “Social security,” “Older adult care service,” “Health support,” and “Emotional comfort.” Using Zhejiang Province as the research subject, real data from 2010 to 2019 were collected for empirical research. The findings of this study are as follows:

Regarding the supply capacity of older adult care services, it exhibited an overall upward trend in Zhejiang Province throughout the observation period. Despite notable improvements in the fairness of spatial distribution over time, spatial differences persist. Specifically, in 2019, the Northern, Southeastern, and Southwestern regions of Zhejiang still displayed a discernible gradient effect in the spatial distribution of older adult care service supply, indicating characteristics of bipolar or multipolar differentiation. Supplementary analysis employing the Dagum Gini coefficient supports these findings, implying the untapped potential for further enhancing the spatial distribution of older adult care services within the province. Attention should be directed toward underserved mountainous areas and island counties in Southeastern Zhejiang.

Over the observation period, the supply capacity of older adult care services in Zhejiang Province demonstrated a significant evolutionary trend from low to high levels, with the possibility of leapfrog evolution, albeit with a low probability value. Notably, geographical location exerted a substantial influence on the dynamic transfer of different types of older adult care service supply capacity, with areas exhibiting high supply capacity positively impacting neighboring regions, indicative of a pronounced club convergence phenomenon.

The aforementioned research findings support the assertions of other authors that China’s overall older adult service supply capacity has been on the rise, owing to the country’s rapid economic development and increased government emphasis on fundamental public services such as healthcare, care services, social insurance, and public culture and sports in recent years. Simultaneously, the spatial disparity in the supply of older adult care services has also diminished (26, 73–78). Furthermore, we conducted a thorough analysis of factors influencing the differences in supply capacity and spatial distribution of older adult care services. Our conclusion highlights the significance of economic development, urbanization rate, transportation capacity, level of openness, and the proportion of employees in the tertiary industry as key influencing factors. These novel findings have significant implications and serve as valuable references for policy formulation in developing countries within the Asia-Pacific region and beyond, aiming to enhance the supply capacity of older adult care services and optimize the spatial distribution of resources in this domain.

### 5.2. Suggestions

Based on the findings, this study proposes the following policy recommendations:

First, it is suggested that the government and relevant departments in Zhejiang Province effectively play the role of linking regions, enhancing the positive spillover effect of regions with a strong supply of older adult services. In the context of establishing a demonstration zone of common prosperity, promoting “Healthy Zhejiang,” and creating a “Zhejiang recreation and health” initiative, the government should strive to adopt a “shared prosperity, sharing, and collaboration” concept between the advanced and relatively less advanced regions in terms of older adult care services. This concept should be informed by modern information technologies, such as 5G, the Internet of Things, artificial intelligence, and meta-universe, to facilitate cooperation and exchange between regions in the areas of older adult medical equipment, older adult technology platforms, older adult service personnel, older adult service facilities, older adult business models, older adult management concepts, older adult innovation achievements, and older adult operational mechanisms. By optimizing the interactive communication and precise support mechanisms for older adult care services, the radiation-driven effect of regions with a strong supply of older adult services can be fully leveraged to improve the spatial allocation of older adult care resources and ensure the balanced development of older adult care services across Zhejiang Province.

Second, it is recommended to explore the new urbanization construction path in light of the concept of common prosperity in the new era and enhance the quality and level of urban and rural older adult service supply through high-level urbanization construction and high-quality economic development. By the end of 2021, the urbanization rate of the resident population in Zhejiang Province was 72.7%, ranking first in the country. In the new historical era of “modernization for all people’s common prosperity,” Zhejiang should leverage the strong resource-gathering and radiative effects of its three leading cities—Hangzhou, Ningbo, and Wenzhou—to narrow economic disparities among cities within the province. To enhance the energy level and resilience of economic development, the government at all levels must optimize the innovation and entrepreneurship environment, promote the construction of a digital economy, and accelerate the digital transformation of industries. The implementation of the “Fourteenth Five-Year Plan” for the development of new-type urbanization in Zhejiang Province should be the government’s focus, continuously improving the relevant supporting policies in the new-type urbanization construction process. The government should prioritize “people-oriented” urbanization and further equalize urban and rural public services. Regarding old-age security, it is recommended to develop a multi-level, multi-pillar, and comprehensive old-age insurance system to break the institutional barriers that hinder the connection between urban and rural old-age insurance, and thus improve the old-age security support capacity of urban workers.

Third, it is essential to address the imbalance between the supply and demand of transportation infrastructure in the province and expedite the construction of transportation infrastructure in underdeveloped regions in the southwest mountainous areas and southeast of Zhejiang, including the Zhoushan islands. It is also necessary to optimize the spatial configuration of transportation networks and to fully leverage the role of transportation infrastructure in enhancing and supporting older adult services in rural and remote areas. This would ultimately achieve the comprehensive development of the entire province. On the one hand, efforts should be made to expand effective investment in transportation. Emphasis should be placed on the balanced development of urban and rural areas and the continued implementation of the “Internal Smooth Outreach” action plan. Transportation infrastructure in mountainous areas, islands, old revolutionary areas, and ethnic areas should be improved and upgraded to provide older adult services in remote regions such as rural areas and islands. To achieve this, a five-level, efficient, interconnected, and interoperable transportation network should be established, from the province to the city, county, township, and village levels. The aim is to form a virtuous cycle where the demand for older adult services can be met, older adult resources can be accessed, and older adult services can be transferred, thereby promoting the overall development of the province. On the other hand, it is recommended to fully implement the “14th Five-Year Plan for the Development of Digital Transportation” and to vigorously develop a digitalized, intelligent, and equitable public transportation system. The competent transportation department of Zhejiang Province should take into account the development realities of different regions and promote the construction of intelligent rail, road, and waterway transportation networks that are tailored to local conditions, taking advantage of the network effects of intelligent transportation. Additionally, efforts should be made to enhance connections and coordination between various regions, including medical service consortia, digital health care consortia, community older adult care communities, remote medical service centers, and other older adult service platforms, to facilitate the smooth delivery of older adult care services and medical security resources in both directions.

Fourth, in the new period, it is suggested that Zhejiang Province optimize its industrial structure and expand its global reach, with a focus on the high-quality development of basic public services; strengthen the top-level design of the older adult service industry by introducing a comprehensive, collaborative, and efficient policy mechanism to promote the standardization, institutionalization, and quality development of foreign investment in the industry; and increase investment in the older adult service industry by including key areas such as intelligent healthcare product research and development, older adult care services, professional education related to older adult care services, and the aging and barrier-free renovation of public facilities in the “Encouraged Foreign Investment Industry Directory (2022 Edition).” This will help to set investment standards and stimulate foreign investment in the industry. To advance the institutional mechanism of older adult services, it is necessary to explore the integration and development mode of both foreign-funded and local older adult care institutions. Efforts should be made to cultivate and incubate innovative older adult service platforms that incorporate distinctive features, strong branding, digitalization, and wisdom to enhance the quality of the older adult service supply. To improve the quality of older adult care services, it is imperative to adopt a synergistic development mechanism that combines the introduction of foreign-trained experts in older adult care with the cultivation of domestic professionals. This can be achieved through strategies such as contracting and hiring foreign experts to provide guidance and instruction on the older adult care business to various older adult care facilities, including nursing homes, senior apartments, and communities, as well as to colleges and universities specializing in healthcare. By integrating the expertise of international specialists with the skills and knowledge of domestic professionals, it is possible to enhance the professionalism, rationalization, and standardization of the older adult care workforce.

## 6. Limitations and future research

The evaluation index system developed in this study to assess the supply capacity of older adult care services is not without limitations, primarily due to data availability and constraints of time and space. Moreover, the data collection presents significant challenges, which in turn greatly hinders the establishment of appropriate instrumental variables to address the endogeneity issue concerning the “causal factors.” We hope that future research endeavors can address this limitation and provide remedies for the aforementioned challenges. Moreover, the rapid advancement of the digital economy and changing demographic characteristics will result in further diversification and personalization of demand for older adult care services. Consequently, the indicators used in this research to measure supply capacity require dynamic updates to capture these evolving trends accurately. Furthermore, the kernel density estimation and Markov chain methods employed in this study have their limitations. While they can illustrate the evolution trend of older adult service supply, they cannot predict long-term trends. To address this, future research should explore the utilization of big data to collect comprehensive information on the supply capacity of older adult care services. By integrating advanced machine learning techniques such as deep learning and data mining, more efficient predictive models can be developed to anticipate the long-term evolution trends in the supply capacity of older adult care services. This will provide a foundation for formulating more scientifically grounded and targeted policies related to older adult care services, resource allocation, and other pertinent aspects.

## Data availability statement

The original contributions presented in the study are included in the article/supplementary material, further inquiries can be directed to the corresponding author.

## Author contributions

HJ contributed to the conception and study design, interpreted the results, and drafted the manuscript. HJ and YY contributed to acquisition of data, analysis, and interpretation of data. All authors contributed to the article and approved the submitted version.
